# Effects of Solid-State Fermentation by *Eurotium cristatum* on the Metabolic Profile of *Angelica dahurica*

**DOI:** 10.3390/foods15071238

**Published:** 2026-04-04

**Authors:** Yuhe Feng, Ailing Chen, Kaiyao Chen, Li Zeng, Xu Ran

**Affiliations:** College of Biomass Science and Engineering, Sichuan University, Chengdu 610065, China; 2022223080056@stu.scu.edu.cn (Y.F.);

**Keywords:** *Angelica dahurica*, *Eurotium cristatum*, solid-state fermentation, volatile metabolites, metabolomics

## Abstract

In this study, *Eurotium cristatum* was used for the solid-state fermentation of *Angelica dahurica*, and the dynamic changes in metabolites during fermentation were investigated. The results showed that fermentation markedly altered the volatile metabolite profile, increasing the relative abundance of terpenoids while decreasing that of alcohols and aldehydes. In combination with principal component analysis (PCA), 24 key volatile compounds were screened. Liquid chromatography–mass spectrometry (LC–MS)-based untargeted metabolomics identified 892 differential annotated non-volatile metabolites and temporal clustering analysis was further applied to characterize their changes. The results showed metabolic fluctuations occurred during the initiation and early fermentation stages, during which carbohydrates and nucleotides were consumed. Secondary metabolites accumulated in the early and middle fermentation. Lipid compounds overall increased in the early fermentation but declined in the middle and late fermentation. Kyoto Encyclopedia of Genes and Genomes (KEGG) pathway enrichment analysis identified 12 key metabolic pathways. This work systematically reveals the change pattern of the metabolite composition of *A. dahurica* driven by *E. cristatum* solid-state fermentation, providing a scientific basis for quality improvement and mechanistic studies of fermented *A. dahurica* products.

## 1. Introduction

*Angelica dahurica* is a medicinal and edible plant, and its dried roots are widely used in traditional medicine and in food applications. Previous studies have identified more than 300 chemical constituents in *A. dahurica*, among which coumarins and volatile oils served as the primary sources of bioactive components and are primarily responsible for its flavor profile [1]. Volatile constituents determine the flavor characteristics of *A. dahurica* and directly affect sensory quality. Hu et al. [2] have identified and evaluated volatile components and key aroma compounds in *A. dahurica* roots using various extraction approaches combined with gas chromatography-mass spectrometry (GC-MS), providing a foundation for aroma characterization. The non-volatile composition of *A. dahurica* is highly complex, including amino acids, polysaccharides, lipids, and typical secondary metabolites such as coumarins and alkaloids [3]. For a long time, studies on the non-volatile metabolites of *A. dahurica* have focused on coumarins, mainly through systematic identification and fingerprint characterization using high-performance liquid chromatography and related analytical methods [4]. In recent years, multi-omics studies have also emerged to elucidate furanocoumarin biosynthesis and regulatory mechanisms governing their accumulation [5]. However, comprehensive untargeted metabolomics analysis of the non-volatile metabolome of *A. dahurica* is still scarce.

In recent years, microbial fermentation has been considered an important approach for processing plant raw materials, as it can not only alter sensory attributes but also enhance bioactivity [6]. *Eurotium cristatum* is a dominant fungus in post-fermented teas such as Fu brick tea and exhibits strong metabolic remodeling capacity. Previous studies have shown that *E. cristatum* fermentation can sharply change the volatile composition of tea, generating and enriching characteristic aroma compounds. Extracellular enzymes secreted by this fungus play decisive roles in the formation of some key aroma compounds [7,8,9]. Except tea, *E. cristatum* has also been applied to solid-state fermentation of multiple substrates such as black soybeans and mulberry leaves, where it also significantly alters volatile profiles and functional activities (e.g., antioxidant performance), indicating cross-substrate potential for remodeling flavor and bioactivity composition [10,11].

Notably, *A. dahurica* has a unique pungent fragrance and a bitter flavor, which makes it mostly used as seasoning rather than food. Microbial fermentation can not only enhance flavor but also reshape the metabolic profile, thereby influencing the sensory and application aspects of food. Zhou et al. [12] applied *E. cristatum* to ferment *A. dahurica* and the results showed that fermentation could enhance the antioxidant activity of *A. dahurica*. However, systematic research is still lacking on the dynamic reconstruction of volatile and non-volatile metabolites during fermentation. Given the prominent metabolic remodeling effects of *E. cristatum* in post-fermented tea, we hypothesized that it may also markedly affect both volatile and non-volatile metabolite profiles of *A. dahurica*. Therefore, it is necessary to introduce untargeted metabolomics to elucidate the chemical evolution of *A. dahurica* under *E. cristatum* fermentation at the level of the metabolic network.

Accordingly, this study used *A. dahurica* as the fermentation substrate and established a time-series sampling system for *E. cristatum* solid-state fermentation. Volatile metabolites were identified and profiled using headspace solid-phase microextraction coupled with gas chromatography–mass spectrometry (HS-SPME-GC-MS), and multivariate statistics were applied to screen key marker volatiles distinguishing different fermentation stages. Subsequently, liquid chromatography–mass spectrometry (LC-MS) based untargeted metabolomics was used to systematically characterize non-volatile metabolites. Temporal clustering analysis of differential non-volatile metabolites performed by the Mfuzzy method was applied to explore the dynamical changes in metabolites. Pathway analysis was further used to provide an interpretation of the metabolic changes associated with *E. cristatum* fermentation. By jointly profiling volatile and non-volatile components of fermented *A. dahurica*, this study explores the changes in *A. dahurica* metabolites of fermentation, providing scientific evidence and new insights for fermentation control and quality improvement of fermented *A. dahurica* products.

## 2. Materials and Methods

### 2.1. Chemicals and Reagents

The raw material of *A. dahurica*’s dried roots was obtained from Yongrong High-Tech Co., Ltd. (Suining, Sichuan, China), harvested at the late vegetative stage, sliced, and dried prior to use. *E. cristatum* was purchased as a lyophilized powder from Shangcheng Beina Chuanglian Biotechnology Co., Ltd. (Xinyang, Henan, China, strain number BNCC146563). All other reagents and chemicals used in this study were purchased from Macklin Biochemical Technology Co., Ltd. (Shanghai, China).

### 2.2. Solid-State Fermentation of Angelica dahurica

*E. cristatum* was activated for two generations on potato dextrose agar (PDA) plates. The plates were incubated for 5 days at 28 °C, during which *E. cristatum* produced abundant golden spores on the surface of the medium. Spores were collected using a sterile cell scraper and transferred to a centrifuge tube containing 40 mL sterile water, followed by centrifugation for 10 min (5000 rpm, 4 °C). After removing the supernatant, spores were resuspended in 10 mL sterile water. Spore concentration was determined using a hemocytometer and adjusted to 6 × 10^7^ CFU/mL.

Dried slices of *A. dahurica* were sterilized by autoclaving at 121 °C for 20 min. After cooling to room temperature, 35 mL of the spore suspension was inoculated onto 100 g of sterilized plant material and shaken for 1 min. The mixture was fermented at 28 °C for 18 days in triplicate batches. Samples were collected at multiple time points: day 0 (S0), day 4 (S4), day 8 (S8), day 10 (S10), day 12 (S12), day 14 (S14), day 16 (S16), and day 18 (S18). Samples were freeze-dried at −50 °C for 72 h, ground into powder using a high-speed grinder, and stored at −80 °C to preserve sample quality.

### 2.3. HS-SPME-GC-MS Analysis

The volatile metabolites of fermented *A. dahurica* were analyzed using HS-SPME-GC-MS with modifications based on the method described by [13], 0.5 g of the sample was placed in a 15 mL headspace vial. An SPME fiber (DVB/CAR/PDMS, 50/30 μm; Supelco, Bellefonte, PA, USA) was exposed to the headspace, and adsorption was performed at 80 °C for 35 min. The fiber was then inserted into the GC-MS inlet and thermally desorbed for 10 min before analysis.

The volatile compounds were detected using a gas chromatography–mass spectrometry system (GCMS-QP2010SE, Shimadzu, Kyoto, Japan) equipped with a VF-WAX capillary column (60 m × 0.25 mm × 0.25 μm). The initial column temperature was 40 °C (held for 1 min), then increased to 180 °C at 5 °C/min (held for 1 min), and further increased to 240 °C at 3 °C/min (held for 6 min). Injections were carried out in splitless mode with the gasification chamber temperature set at 240 °C. Helium was used as the carrier gas at a flow rate of 1 mL/min. Mass spectrometer parameters included an electron bombardment ion source (EI) voltage of 70 eV, an ion source temperature of 240 °C, an interface temperature of 240 °C, and a scanning range of *m*/*z* 35–500 amu. Volatile compounds were characterized in combination with similarity index (SI) and retention index (RI) which was determined using n-alkanes (C7–C30). The relative content of each compound was determined using peak area normalization.

### 2.4. LC-MS Analysis

The non-volatile metabolites in samples (50 mg) were extracted with 400 μL extract solution (Vmethanol:Vwater = 4:1, with 0.02 mg/mL L-2-chlorophenylalanine as the internal standard) at 5 °C for 30 min in a SBL-10TD ultrasonic bath (Xinzhi Biotechnology, Ningbo, Zhejiang, China) after homogenization by a Wonbio-96c freezing grinder (Wanbo Biotechnology, Shanghai, China) at −10 °C for 6 min. Then, sample extracts were incubated at −20 °C for 30 min and centrifuged at 13,000 r/min and 4 °C for 15 min by Centrifuge 5430R (Eppendorf, Hamburg, Germany). The final supernatant was filtered through a 0.22 μm membrane for further analysis. Quality control (QC) samples were prepared by pooling 20 μL of supernatant from each sample.

LC-MS analysis was performed using a UHPLC-Q Exactive system (Thermo Fisher Scientific, Waltham, MA, USA). Chromatographic separation was achieved using an ACQUITY UPLC HSS T3 column (100 mm × 2.1 mm, 1.8 μm; Waters Corporation, Milford, MA, USA). The mobile phase solvent A was 2% acetonitrile in water containing 0.1% formic acid, and solvent B was acetonitrile containing 0.1% formic acid. The injection volume was 3 μL, and the column temperature was maintained at 40 °C. The gradient program is provided in Table S1a. After electrospray ionization, MS signals were acquired in both ESI+ and ESI− modes. The detailed mass spectrometry parameters are listed in Table S1b. During instrumental analysis, one QC sample was inserted after every eight analytical samples to monitor the stability of the detection process.

After data acquisition by LC-MS, mass spectrometric feature extraction was performed by using Progenesis QI (Corporation, Milford, MA, USA). The metabolites annotation was performed by matching the spectral data against public databases (e.g., KEGG, https://www.kegg.jp/ (accessed on 5 January 2026) and HMDB, http://www.hmdb.ca/ (accessed on 5 January 2026)) and the Majorbio in-house database. Subsequently, data analysis was performed using the Majorbio Cloud Platform (https://cloud.majorbio.com), which included multivariate statistical analysis and pathway enrichment analysis to interpret metabolomic profiles. The data matrix underwent rigorous pre-processing: variables containing non-zero values in at least 80% of the samples were retained, followed by the minimum value imputation. Furthermore, variables with a relative standard deviation (RSD) > 30% in the quality control (QC) samples were excluded from subsequent statistical analyses.

### 2.5. Statistical Analysis and Visualization

All experiments were conducted in triplicate, data were presented as mean ± standard deviation. Preliminary statistical analysis was performed using Microsoft Excel (version 2021, Microsoft Corporation, Redmond, WA, USA). Metaboanalyst 6.0 was employed for one-way analysis of variance (ANOVA), principal components analysis (PCA), partial least squares discriminant analysis (PLS-DA) and heat maps. Statistical significance was determined based on the *p*-value < 0.05. Data visualizations were created using Origin 2024 (OriginLab Corporation, Hampton, MA, USA).

## 3. Results and Discussion

### 3.1. Analysis of Volatile Metabolites

#### 3.1.1. Overall Evaluation of Volatile Metabolites in the Fermentation Process

**Figure 1:**
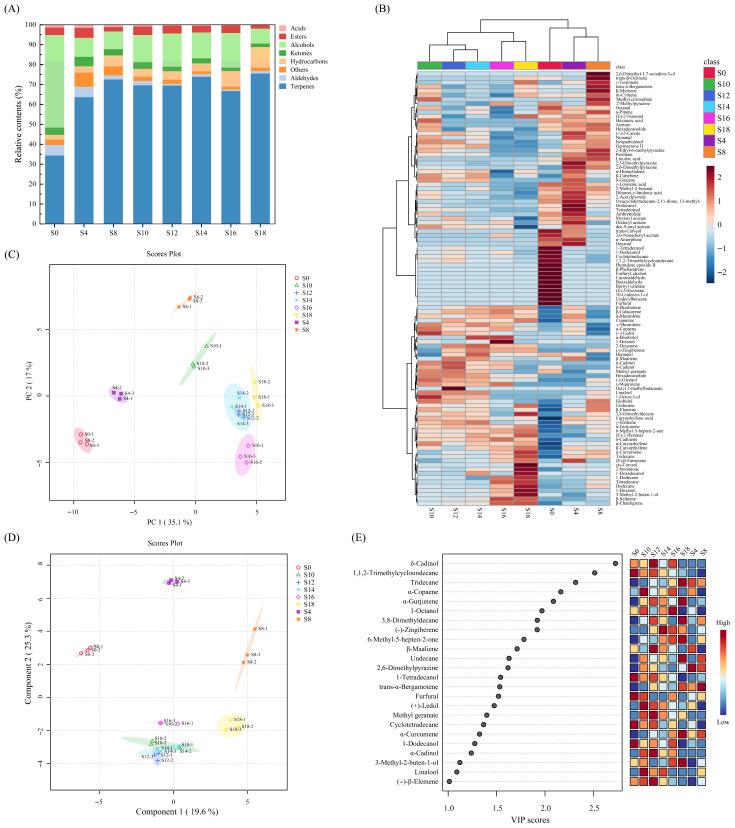
Changes in the volatile metabolite profile of *Angelica dahurica* during fermentation. Changes in relative contents (**A**) and hierarchical clustering heatmap (**B**) of volatile metabolites during fermentation. PCA score plot (**C**) and PLS-DA score plot (**D**) of volatile metabolites during fermentation. VIP (Variable importance in projection) from the PLS-DA model (**E**). S0, S4, S8, S10, S12, S14, S16 and S18 mean days 0, 4, 8, 10, 12, 14, 16 and 18 of fermentation, respectively.

To characterize changes in volatile metabolites during *E. cristatum* fermentation of *A. dahurica*, HS-SPME-GC-MS was applied. In total, 102 volatile metabolites were identified across all sample groups, including 45 terpenoids, 12 aldehydes, 11 hydrocarbons, 10 esters, 9 alcohols, 5 ketones, 4 acids, and 6 compounds in other categories (Table S2). Figure 1A illustrates the relative changes in volatile metabolite concentrations during fermentation, indicating that *E. cristatum* fermentation caused substantial changes in volatile composition. Figure 1B presents a heatmap of volatile metabolites profiles during fermentation and cluster analysis, dividing samples into four stages: initiation (S0), early fermentation (S4 and S8), middle fermentation (S10, S12, and S14), and late fermentation (S16 and S18), indicating stage-specific shifts in volatile composition during fermentation.

Overall, terpenoids were the predominant volatile compounds in all samples, consistent with previous reports that *A. dahurica* essential oil is mainly composed of monoterpenes and sesquiterpenes [2,14]. In the S0 sample, alcohols accounted for 46.47% of total volatiles, whereas terpenoids accounted for 34.38%. In fermentation progression, terpenoids rapidly became dominant (>60% at all time points, reaching 75.47% at S18), with particularly increases in β-elemene, β-caryophyllene, and γ-elemene. Conversely, some terpenoids initially present (e.g., α-pinene and trans-carveol) gradually decreased and eventually became undetectable. This agrees with prior observations that *E. cristatum*-involved fermentation can markedly remodel volatile profiles and shift abundance distributions of terpene-related compounds [7,15]. In addition, 11 new terpenoids emerged during fermentation, suggesting that fermentation may be associated with the release and structural transformation of terpenoid compounds [16]. Based on reported aroma descriptors of detected compounds (Table S2), the increased relative contribution of terpenoids may be associated with enhanced herbaceous, woody, and spicy sensory notes.

In contrast to terpenoids, alcohols decreased sharply during early fermentation, dropping from 46.47% in S0 to 8.90% in S8. Alcohols then slightly rebounded in the middle fermentation and decreased again to 7.49% by the end. In particular, 1-dodecanol and 1-tetradecanol decreased markedly, which may alleviate the waxy and fatty base note of *A. dahurica* (Table S2). Meanwhile, typical fungal volatile metabolites with mushroom odors, such as 1-octen-3-ol [17], appeared during middle and late fermentation and then disappeared, suggesting that fatty acid metabolism changes may occur during middle fermentation, possibly contributing to the formation of compounds such as 1-octen-3-ol [18,19].

Acids accounted for a small fraction overall. Their relative content was 1.41% in S0, then decreased significantly as fermentation proceeded to 0.19% in S18. γ-Linolenic acid accounted for 1.21% in S0 as the major acid component and remained the only detectable acid in the late fermentation. It should be noted that relative abundances in this study were normalized to the total peak area at each time point; thus, the decrease in acid proportion may reflect actual oxidative degradation of acids [10,11] and a proportional dilution effect due to substantial accumulation of other classes such as terpenoids during fermentation.

Aldehydes showed an overall continuous decrease, from 5.29% in S0 to 1.33% in S18. Decanal, dodecanal, and furfural decreased significantly and disappeared in the middle and late fermentation. By contrast, (E)-2-hexenal and (E)-2-nonenal accumulated in middle and late fermentation, indicating a reconstruction of aldehyde composition. This phenomenon may be associated with microbial conversion of certain aldehydes via further reduction to alcohols [20,21] or oxidation to acids [22].

Ketones exhibited a transient increase to 4.74% in S4, followed by a gradual decrease to 1.71% by the end of fermentation, mainly due to the decline of compounds such as 13-methyl-oxacyclotetradecane-2,11-dione at late stages. Meanwhile, low molecular weight ketones such as 2-nonanone and 6-methyl-5-hepten-2-one emerged and increased during fermentation, suggesting that fermentation may involve oxidative cleavage of lipids [23,24].

Esters peaked at 5.01% in S4 and then fluctuated slightly within 1.75–4.25%. Esters were dominated by long-chain fatty acid acetates, with dodecyl acetate as a major component. In addition, multiple esters present in the initial material decreased significantly and disappeared at late fermentation (e.g., dec-9-enyl acetate, myristyl acetate, and bornyl valerate), which may be related to ester hydrolysis during fermentation [25].

Overall, fermentation significantly altered the composition of volatile metabolites in *A. dahurica*, shifting the system from an initiation pattern in which alcohols and terpenoids jointly dominated to a late-fermentation profile primarily dominated by terpenoids with smaller contributions from alcohols, hydrocarbons, esters, and ketones, indicating a clear reshaping effect on aroma style.

#### 3.1.2. Analysis of PCA and PLS-DA of Volatile Metabolites

To better evaluate differences in volatiles, PCA and PLS-DA were performed for samples collected at different fermentation days. As shown in Figure 1C, the first two principal components explained 35.1% and 17.0% of the total variance, respectively, and within-group reproducibility was good across time points. S0 was clearly separated from fermented samples. As fermentation progressed, samples exhibited continuous evolution over time in their volatile profiles. Overlap was observed between S14 and S12, indicating similar volatile profiles in those samples. Based on these results, the fermentation process was divided into four distinct stages, which were consistent with the cluster analysis results. The loading plot (Figure S1A) showed that PC1 was mainly driven by β-selinene, dodecanal, decanal, (E)-2-hexenal, etc., whereas PC2 was driven by methyl citronellate, β-maaliene, heptanal, α-copaene, etc.

PLS-DA is widely used for classification in metabolomics data analysis. A PLS-DA model was further established (Figure 1D) and validated (Figure S1C,D), showing satisfactory discrimination among samples from different fermentation stages, as supported by cross-validation and permutation testing. Based on variable importance in projection (VIP) values and univariate tests, significantly differential volatiles were screened (VIP > 1, *p* < 0.05), yielding 24 key marker compounds (Figure 1E). These differential volatiles exhibited clear stage-specific patterns: one group showed relatively high proportions in initiation or early fermentation and decreased subsequently (e.g., 1,1,2-trimethylcycloundecane, 1-tetradecanol, and furfural), whereas another group increased in middle and late fermentation and drove separation of late fermentation samples, consisting mainly of sesquiterpenes and derivatives (e.g., α-gurjunene, δ-cadinol, and (-)-zingiberene). Notably, δ-cadinol and α-gurjunene reached peak levels in middle and late fermentation, suggesting that *E. cristatum* fermentation may involve terpene release and transformation, thereby driving stage-specific evolution of volatile characteristics.

### 3.2. Untargeted Metabolomic Analysis of Non-Volatile Metabolites

In this study, untargeted metabolomics was employed to systematically characterize *A. dahurica* during fermentation. Previous metabolomics research on the drying process of *A. dahurica* has reported that nearly one thousand metabolites can be detected, mainly distributed among more than ten major categories such as amino acids and derivatives, phenolic acids, coumarins, lipids, nucleosides and derivatives, etc. [26].

#### 3.2.1. Overall Composition and Phytochemical Classification

**Figure 2:**
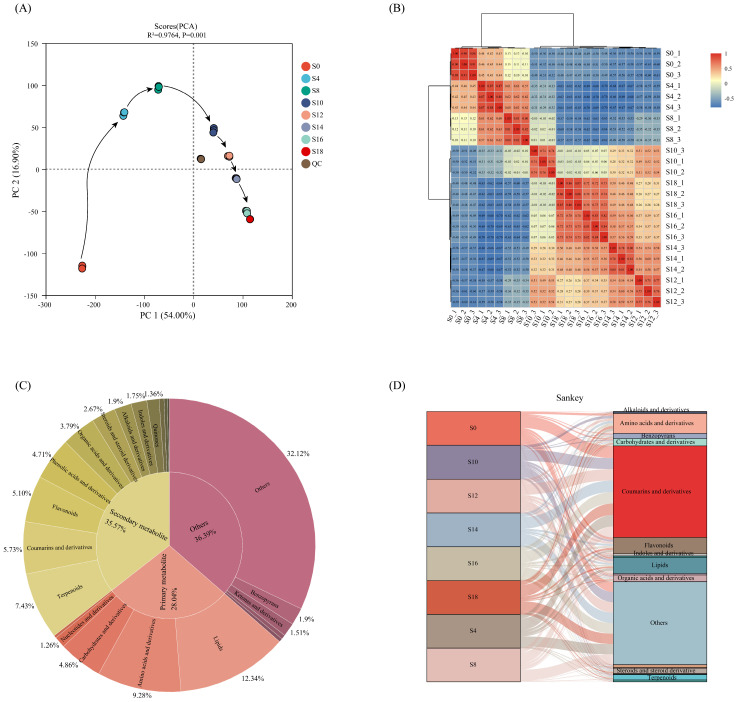
Overview of metabolomics of *Angelica dahurica* fermentation. PCA scores plot (**A**) and correlation heatmap (**B**) of non-volatile metabolites across different samples. Substance classification diagram (**C**) and sankey diagram (**D**) of non-volatile metabolites during fermentation. S0, S4, S8, S10, S12, S14, S16 and S18 mean days 0, 4, 8, 10, 12, 14, 16 and 18 of fermentation, respectively.

Quality control (QC) samples showed high clustering in the PCA plot (Figure 2A), indicating good analytical stability. A total of 2070 metabolite features were obtained. After data preprocessing (feature filtering and missing value imputation), 2058 features were retained, and data drift was markedly reduced (Figure S2A).

PCA results demonstrated clear differences in metabolite profiles across fermentation time (Figure 2A). PC1 and PC2 explained 55.40% and 17.40% of variance, respectively, and samples displayed a clear temporal trajectory, indicating time-dependent reshaping of the *A. dahurica* metabolome. In addition, Spearman’s correlation coefficients were calculated based on detected metabolites and used to generate a sample correlation heatmap (Figure 2B). Correlation values closer to 1 indicate higher similarity in metabolite composition and abundance. Within-group correlation coefficients were generally >0.7; S0, S4, and S8 samples were relatively close to each other, while S10, S12, S14, S16, and S18 samples were also relatively close. These results indicate that fermentation time is a major driver of metabolic profile changes. Based on these results, the fermentation process, as reflected by non-volatile metabolites, could also be divided into four distinct stages, in agreement with the clustering of volatile metabolites.

A total of 1244 annotated metabolites were identified in positive ion mode and 814 metabolites in negative ion mode, yielding 2058 annotated metabolites overall. Based on phytochemical characteristics, metabolites were categorized into primary metabolites, secondary metabolites, and others (Figure 2C). Primary metabolites included amino acids and derivatives (191), carbohydrates and derivatives (100), lipids (254), nucleotides and derivatives (26), and vitamins (6). Secondary metabolites included terpenoids (153), coumarins and derivatives (118), flavonoids (105), phenolic acids and derivatives (97), organic acids and derivatives (78), steroids and derivatives (55), alkaloids and derivatives (39), indoles and derivatives (36), quinones (28), stilbenes (11), lignans and derivatives (8), and tannins (4). Other metabolites included benzopyrans (39), ketones and derivatives (31), aldehydes and derivatives (10), aldehydes and derivatives (8), and other metabolites (661). To more intuitively illustrate how different metabolite classes changed during fermentation, a Sankey diagram was constructed to visualize time-dependent changes in the relative abundance of each class (Figure 2D). Overall, lipid proportions increased first and then decreased; carbohydrates and derivatives and nucleotides and derivatives decreased over time; amino acids and derivatives increased over time. Coumarins and derivatives represented the largest fraction and fluctuated during fermentation. Linear furanocoumarins were prominent, including imperatorin, isoimperatorin, methoxsalen, bergapten, bergaptol, isopimpinellin, and xanthotoxol. Related compounds such as oxypeucedanin, (R)-heraclenol, marmesin, and columbianetin were also detected. In addition, the proportions of steroids and derivatives, terpenoids, alkaloids and derivatives, vitamins, and quinones gradually increased with fermentation. These results indicate active decomposition and remodeling of primary metabolism during *E. cristatum* fermentation, accompanied by enhancement of secondary metabolic pathways, which may contribute to enrichment and activation of characteristic components of *A. dahurica*.

#### 3.2.2. Differential Non-Volatile Metabolites Analysis

**Figure 3:**
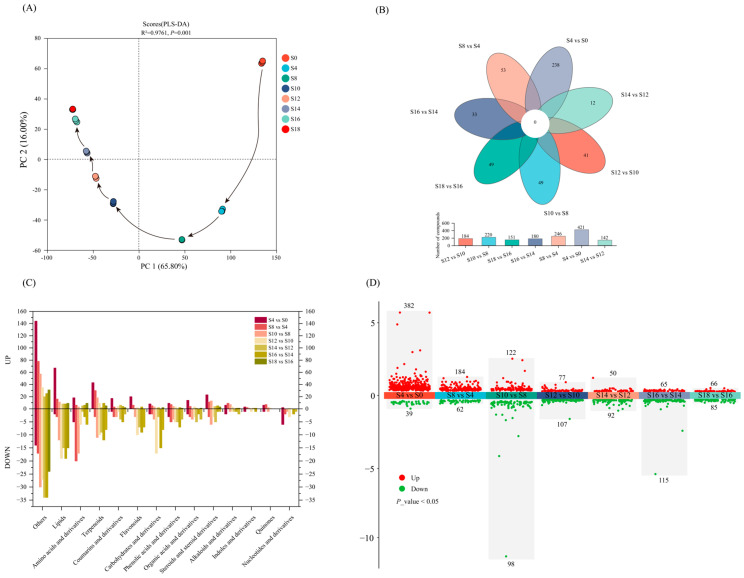
Analysis of differential non-volatile metabolites. PLS-DA scores plot of non-volatile metabolites during fermentation of *Angelica dahurica* (**A**). Venn diagram (**B**) and classification charts (**C**) of differential metabolites from seven pairwise comparisons. Multiple differential volcano plot for the seven pairwise comparisons of differential metabolites. (**D**) S0, S4, S8, S10, S12, S14, S16 and S18 mean days 0, 4, 8, 10, 12, 14, 16 and 18 of fermentation, respectively.

PLS-DA model was used to describe changes in non-volatile metabolites during fermentation (Figure 3A). Cross-validation indicated that the model was stable, reliable, and had strong predictive capability (R^2^ = 0.994, Q^2^ = 0.943; Figure S3). PLS-DA results showed clear separation among samples at different fermentation times, confirming that *E. cristatum* fermentation significantly affected the metabolite profile of *A. dahurica*.

To further analyze relationships between non-volatile metabolites and fermentation time and to screen significant differential metabolites, orthogonal partial least squares-discriminant analysis (OPLS-DA) was applied. OPLS-DA models were built for samples at adjacent fermentation time points (S4 vs. S0, S8 vs. S4, S10 vs. S8, S12 vs. S10, S14 vs. S12, S16 vs. S14, and S18 vs. S16) to identify key differential metabolites. The OPLS-DA models clearly separated paired groups, and permutation tests yielded R^2^Y and Q^2^ values > 0.9 (Figure S3A–G), indicating acceptable model performance in permutation testing. Multivariate analyses using PLS-DA and OPLS-DA confirmed statistically significant differences in non-volatile metabolites among samples at different fermentation times. Using VIP > 1.5 and FDR < 0.05 as the screening threshold, 892 differential metabolites were obtained across the eight sample groups (Table S4), mainly including lipids and lipid-like molecules, organic acids and derivatives, phenylpropanoids and polyketides, organic oxygen compounds, organoheterocyclic compounds, and benzenoids. A Venn diagram of differential metabolites from pairwise comparisons is shown in Figure 3B. No shared differential metabolites were observed among the comparison sets. Moreover, S4 vs. S0 had the highest number of unique differential metabolites, whereas S14 vs. S12 had the fewest, indicating that the early fermentation stage is crucial for *A. dahurica* fermentation. Differential metabolites between adjacent time points were annotated by phytochemical class (Figure 3C), and multiple volcano plots were generated (Figure 3D). The number of significant differential metabolites varied markedly across comparisons and decreased progressively with fermentation, stabilizing at later stages: 421 (S4 vs. S0), 246 (S8 vs. S4), 220 (S10 vs. S8), 184 (S12 vs. S10), 142 (S14 vs. S12), 180 (S16 vs. S14), and 151 (S18 vs. S16). These comparisons yielded 382, 184, 122, 77, 50, 65, and 66 up-regulated metabolites, and 39, 62, 98, 107, 92, 115, and 85 down-regulated metabolites, respectively. Lipids and lipid-like molecules, amino acids and derivatives, terpenoids, and other classes were the major types among both up- and down-regulated metabolites. In early fermentation, the number of up-regulated metabolites exceeded that of down-regulated metabolites, reflecting increased overall metabolic activity and continuous accumulation of new components during early fermentation.

#### 3.2.3. Temporal Clustering Analysis

**Figure 4:**
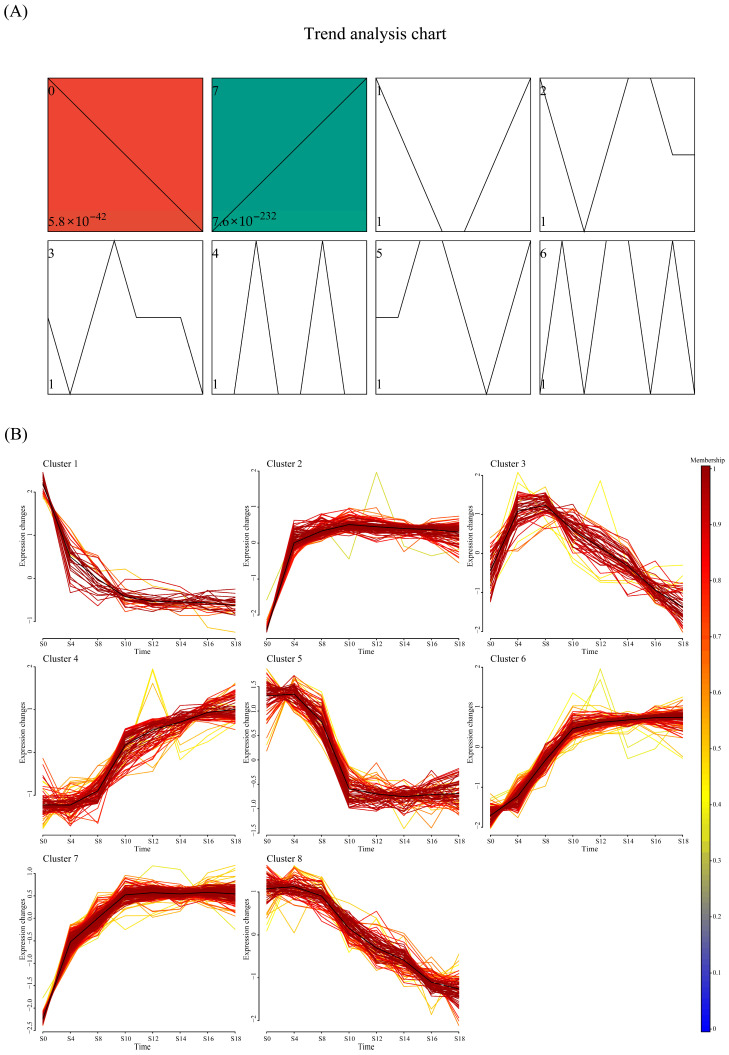
Time series analysis. STEM clustering algorithm analysis of the dynamic alteration of the differential metabolites in *Angelica dahurica* during fermentation (**A**). Clustering analysis using the fuzzy c-means algorithm (Mfuzz) based on differential metabolites from significant change trends by STEM analysis (**B**). S0, S4, S8, S10, S12, S14, S16 and S18 mean days 0, 4, 8, 10, 12, 14, 16 and 18 of fermentation, respectively.

Since pairwise comparisons alone cannot comprehensively summarize time-dependent trends, short time-series expression miner (STEM) analysis was performed based on significant differential metabolites obtained from OPLS-DA. As shown in Figure 4A, two significantly changing temporal trends were identified (q < 0.05), corresponding to an overall increasing trend (455 metabolites) and an overall decreasing trend (228 metabolites). To examine consistency within these trends, Mfuzz trend analysis was further performed. As shown in Figure 4B, 683 differential metabolites were classified into eight temporal patterns, and phytochemical classification was conducted for each cluster (Table S5). Clusters 1 through 8 contained 28, 100, 50, 85, 69, 113, 157, and 81 metabolites, respectively.

Clusters 1, 5, and 8 showed a decreasing trend. Cluster 1 was primarily composed of carbohydrates and nucleosides, including trehalose, sucrose, adenosine, deoxyadenosine, and guanine. Cluster 5 contained 15 amino acids and derivatives, predominantly dipeptides rich in leucine (Leu), isoleucine (Ile), valine (Val), and phenylalanine (Phe), suggesting that during the early fermentation, dipeptide nitrogen was preferentially assimilated [27]. Clusters 1 and 5 suggest that during the initiation and early fermentation, *E. cristatum* proliferated rapidly and utilized these substrates (carbon sources and nucleosides) for energy production and nucleic acid synthesis. Cluster 8 contained various glycosides, including α-D-glucose, hydroxytyrosol 1-O-glucoside, and p-coumaric acid glucoside, suggesting that glycoside hydrolysis may occur during fermentation [28].

Clusters 2, 6, and 7 showed rapid accumulation of metabolites from day 0 to day 10 of fermentation, followed by stabilization. Cluster 4 displayed a continuously increasing trend throughout the fermentation process. The metabolites clustered in clusters 2, 4, 6, and 7 were mainly lipids, terpenoids, coumarins and derivatives, flavonoids, steroids and derivatives. These secondary metabolites are important active components in *A. dahurica* [29]. Additionally, Cluster 7 also contained 12 amino acid derivatives, including homostachydrine, N-methyl-L-proline, γ-glutamyl-L-putrescine, N-succinyl-2,6-diaminopimelate, Trp-Thr, Trp-Pro, Trp-Thr, Phe-His, indicating that nitrogen metabolism in the initiation and early fermentation stages was significantly impacted.

Cluster 3 peaked early in fermentation and then declined. Cluster 3 contained 19 lipid compounds, including various lysophospholipids (LysoPC, LysoPE, LysoPG, LysoPS), PC types, and unsaturated fatty acids and derivatives. This suggests that more intense membrane lipid metabolism may occur in the early fermentation stages, leading to short-term accumulation of lysophospholipids and fatty acyl intermediates. This pattern may reflect subsequent turnover or redistribution of lipid-related metabolites during middle and late fermentation, possibly involving membrane lipid remodeling and other downstream metabolic processes [30,31].

The temporal clustering analysis indicates that the initiation and early fermentation are critical periods of metabolic change during *E. cristatum* fermentation, during which primary metabolites such as carbohydrates, amino acids, and nucleotides are consumed, while lipid compounds and free secondary metabolites are released. As fermentation progresses into the middle and late fermentation, readily translatable substrates are exhausted, the overall fungal metabolic flux decreases, and the material changes reach a dynamic equilibrium.

#### 3.2.4. Key Metabolic Pathway Analysis

**Figure 5:**
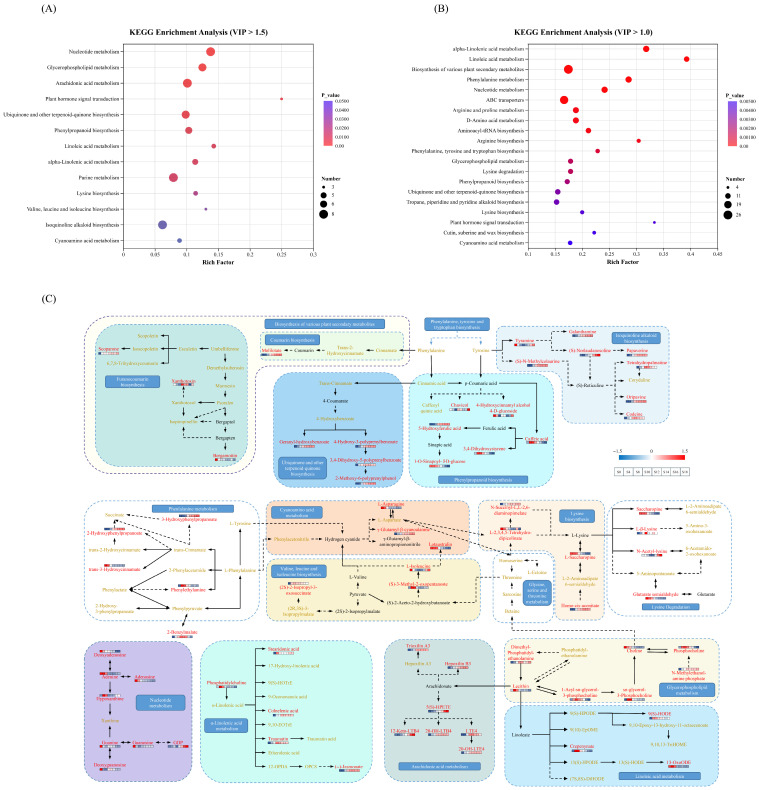
Analysis of key metabolic pathways. KEGG enrichment analysis on the differential metabolite sets (VIP > 1.5 and FDR < 0.05) (**A**) and (VIP > 1 and FDR < 0.05) (**B**), respectively. KEGG-based pathway-module relationships for key differential metabolites of *Angelica dahurica* (**C**). Differential metabolites in metabolic pathways are color-coded: red represents those with VIP > 1.5 and FDR < 0.05, while orange represents those with 1.5 > VIP > 1 and FDR < 0.05. The key metabolic pathway is indicated by the colored background, with 12 pathways in total.

To elucidate metabolic network changes in key differential non-volatile metabolites during *E. cristatum* fermentation of *A. dahurica*, KEGG pathway enrichment analysis was conducted using the 892 differential metabolites obtained by OPLS-DA (VIP > 1.5 and FDR < 0.05; Table S6). In addition, to improve pathway coverage and enhance biological interpretation of the metabolic network, an expanded differential metabolite set (VIP > 1 and FDR < 0.05) was also analyzed (Table S7). As shown in Figure 5A,B, the two enrichment analyses shared 12 overlapping pathways. A key metabolic network diagram was constructed (Figure 5C), presenting global metabolic changes across key KEGG pathways during the 18-day fermentation process.

Enrichment of nucleotide metabolism suggests that enhanced nucleotide metabolic activity during initiation and early fermentation. Nucleotide metabolism not only provides precursors for DNA and RNA synthesis but also supports other metabolic reactions through high-energy nucleotides such as GDP, thereby providing material and energy foundations for subsequent substrate conversion and operation of diverse metabolic networks [32,33].

*E. cristatum* fermentation of *A. dahurica* altered the amino acid metabolic network, with significant enrichment in lysine biosynthesis, valine, leucine and isoleucine biosynthesis, and cyanoamino acid metabolism. In the lysine biosynthesis pathway, the levels of saccharopine and L-2,3,4,5-tetrahydrodipicolinate rapidly declined during fermentation, whereas those of homo-cis-aconitate and N-succinyl-L,L-2,6-diaminopimelate increased significantly. Metabolites in the branched-chain amino acid biosynthesis pathway exhibited complex dynamic trends. The concentrations of (S)-3-methyl-2-oxopentanoate and (2S)-2-isopropyl-3-oxosuccinate first rose and then fell, whereas L-isoleucine displayed an initial decrease followed by a subsequent increase. These pathways are closely associated with microbial growth and metabolite transformation.

Lipid metabolism was significantly concentrated in glycerophospholipid metabolism and polyunsaturated fatty acid (PUFA)-related pathways, including linoleic acid, α-linolenic acid, and arachidonic acid metabolism, suggesting that membrane lipid alterations and PUFA oxidative network remodeling occur during fermentation. Glycerophospholipid metabolism plays a critical role in fungal growth and metabolism [34]. The key phospholipid compounds in this pathway (e.g., phosphatidylcholine and phosphocholine) increased in the early fermentation. These phospholipid-related components serve as precursors to linoleate, α-linolenate, and arachidonate, linking glycerophospholipid metabolism with PUFA-related pathways. In the α-linolenic acid metabolism pathway, various oxidation products increased, and both (−)-jasmonate and its precursor OPDA accumulated during middle fermentation, indicating alterations in α-linolenic acid-derived oxylipin metabolism. Similarly, in the linoleic acid and arachidonic acid metabolism pathways, multiple oxidative derivatives (e.g., 9(S)-HODE, 5(S)-HPETE, hepoxilin B3, 12-keto-LTB4, LTE4) increased in the middle and late fermentation, indicating that enhanced oxidative conversion of unsaturated fatty acids occurs during fermentation.

The phenylpropanoid metabolic pathway was enriched in both free compounds (such as cinnamic acid and caffeic acid) and typical conjugated or derivative compounds (such as caffeoylquinic acid, 5-hydroxyferulic acid, 3,4-dihydroxystyrene, 1-O-sinapoyl-β-D-glucose, and 4-hydroxycinnamyl alcohol 4-O-glucoside). The variations in these compounds suggest that some aromatic compounds may undergo glycoside hydrolysis and subsequent redistribution among modification branches during fermentation [35,36,37].

Plant secondary metabolic pathways were dominated by coumarin and furanocoumarin biosynthesis. Coumarin is an important active constituent of *A. dahurica*. In the furanocoumarin biosynthesis pathway, scopoletin and isoscopoletin were reduced during fermentation, whereas the final product scoparone increased, which may be related to increased O-methylation or enhanced stability of methylated products [38]. In the coumarin biosynthesis pathway, melilotate progressively accumulated as fermentation proceeded, whereas its upstream precursors, trans-cinnamate and 2-hydroxycinnamic acid, both decreased. Thus, the accumulation of melilotate may reflect microbial transformation of metabolites related to hydroxycinnamic acids during fermentation [39,40]. These changes in coumarin-related compounds could impact the bioactivity and quality of the fermented *A. dahurica* product.

The ubiquinone and other terpenoid-quinone biosynthesis pathway was significantly enriched, attributed to a group of key differential metabolites (geranyl-hydroxybenzoate, 4-hydroxy-3-polyprenylbenzoate, 3,4-dihydroxy-5-polyprenylbenzoate, and 2-methoxy-6-polyprenylphenol) whose levels increased during fermentation. Additionally, isoquinoline alkaloid biosynthesis was significantly enriched, associated with various compounds of notable bioactivity [41].

KEGG pathway enrichment analysis indicated that fermentation not only altered primary metabolic networks but also promoted changes in secondary metabolism, collectively shaping the stage-specific succession of the metabolic profile during fermentation. The initial and early fermentation were dominated by substrate-related metabolism (e.g., amino acids and nucleotides) and membrane lipid turnover, whereas the middle and late fermentation were characterized by oxidation of unsaturated fatty acids and sustained accumulation of plant secondary metabolites.

## 4. Conclusions

This study employed HS-SPME-GC-MS and the untargeted metabolomic analyses to investigate the volatile and non-volatile metabolites during the solid-state fermentation of *A. dahurica* by *E. cristatum*. In the volatile metabolite analysis, 102 compounds were detected, of which 24 were identified as key volatile metabolites. These results indicated that the volatile metabolite profile underwent significant stage-dependent changes during fermentation. Terpenes produced during fermentation constituted the main volatile components of the fermented *A. dahurica* and their relative abundance increased significantly, whereas the levels of alcohols and aldehydes decreased. For non-volatile metabolites, a total of 2058 compounds were detected, of which 892 annotated non-volatile metabolites were screened as differential features. The Mfuzz analysis was used to analyze the dynamic changes in these differential metabolites during fermentation. The results showed that in the early fermentation, primary metabolites (e.g., amino acids, nucleotides, and carbohydrates) were primarily consumed and restructured. In contrast, during the early to middle fermentation, secondary metabolites such as terpenes and steroids began to accumulate. Lipid compounds overall increased in the early fermentation but declined in the middle and late fermentation. The associated KEGG metabolic pathways mainly included nucleotide metabolism, amino acid metabolism, PUFA metabolism, and coumarin metabolism.

This study is the first systematic analysis of the effects of *E. cristatum* fermentation on the chemical composition of *A. dahurica*, providing valuable insights into changes in the metabolite profile during solid-state fermentation and offering a theoretical basis for flavor regulation and quality improvement of fermented *A. dahurica* products. Future studies may combine multi-omics approaches to further elucidate the fermentation mechanisms, thereby enabling precise control and process optimization of *A. dahurica* fermentation.

## Data Availability

The original contributions presented in this study are included in the article/Supplementary Materials. Further inquiries can be directed to the corresponding authors.

## Supplementary Materials

The following supporting information can be downloaded at: https://www.mdpi.com/article/10.3390/foods15071238/s1, Figure S1: Validation of the GC-MS multivariate models. Figure S2: Metabolomics data quality and PLS-DA model validation. Figure S3: OPLS-DA models for pairwise comparisons during fermentation. Table S1: Gradient elution program and mass spectrometry conditions. Table S2: Relative content of volatile metabolites in different fermented time of *Angelica dahurica*. Table S3: Differential metabolites screened from all samples by PLS-DA. Table S4: Summary of differential metabolites derived from pairwise comparisons during fermentation. Table S5: Classification statistics of differential metabolites based on MFuzz time-series clustering. Table S6: The significantly enriched KEGG pathways of differential metabolites (VIP > 1.5). Table S7: The significantly enriched KEGG pathways of differential metabolites (VIP > 1.0).
