# Supplementary material for: Effects of Solid-State Fermentation by Eurotium cristatum on the Metabolic Profile of Angelica dahurica
Source: Foods. 2026 Apr 4;15(7):1238. doi: 10.3390/foods15071238 (PMC13073739; doi:10.3390/foods15071238)
Supplement: Supplementary file 1 [file foods-15-01238-s001.zip › foods-4213594-Supplementary_figures_for proofreading.pdf]

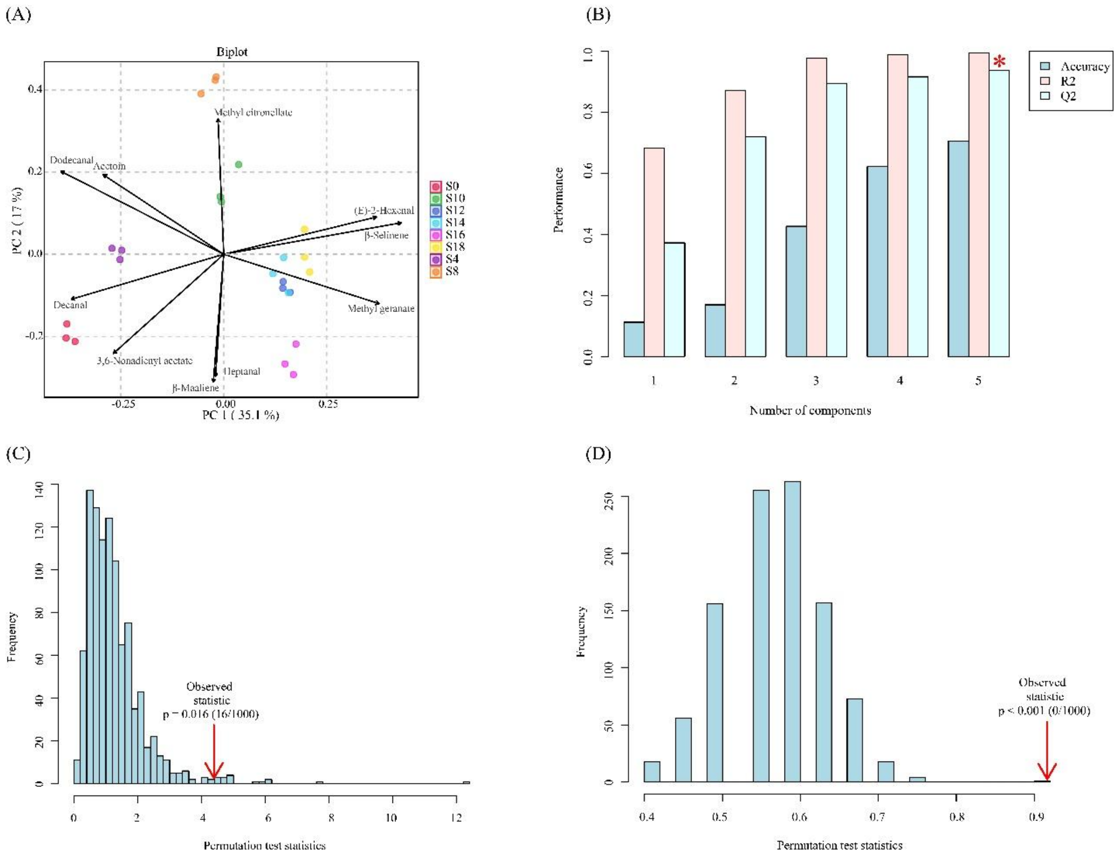

Figure S1. Validation of the GC-MS multivariate models. PCA loadings plot (A). Performance metrics (Accuracy= 0.643, R^2=0.995, Q^2=0.944) of the PLS-DA model from 5-fold cross-validation (B). Results of 1000 permutation tests demonstrating the statistical significance ($p < 0.001$ for training accuracy in C; $p = 0.0016$ for B/W separation distance in D) of the PLS-DA model.

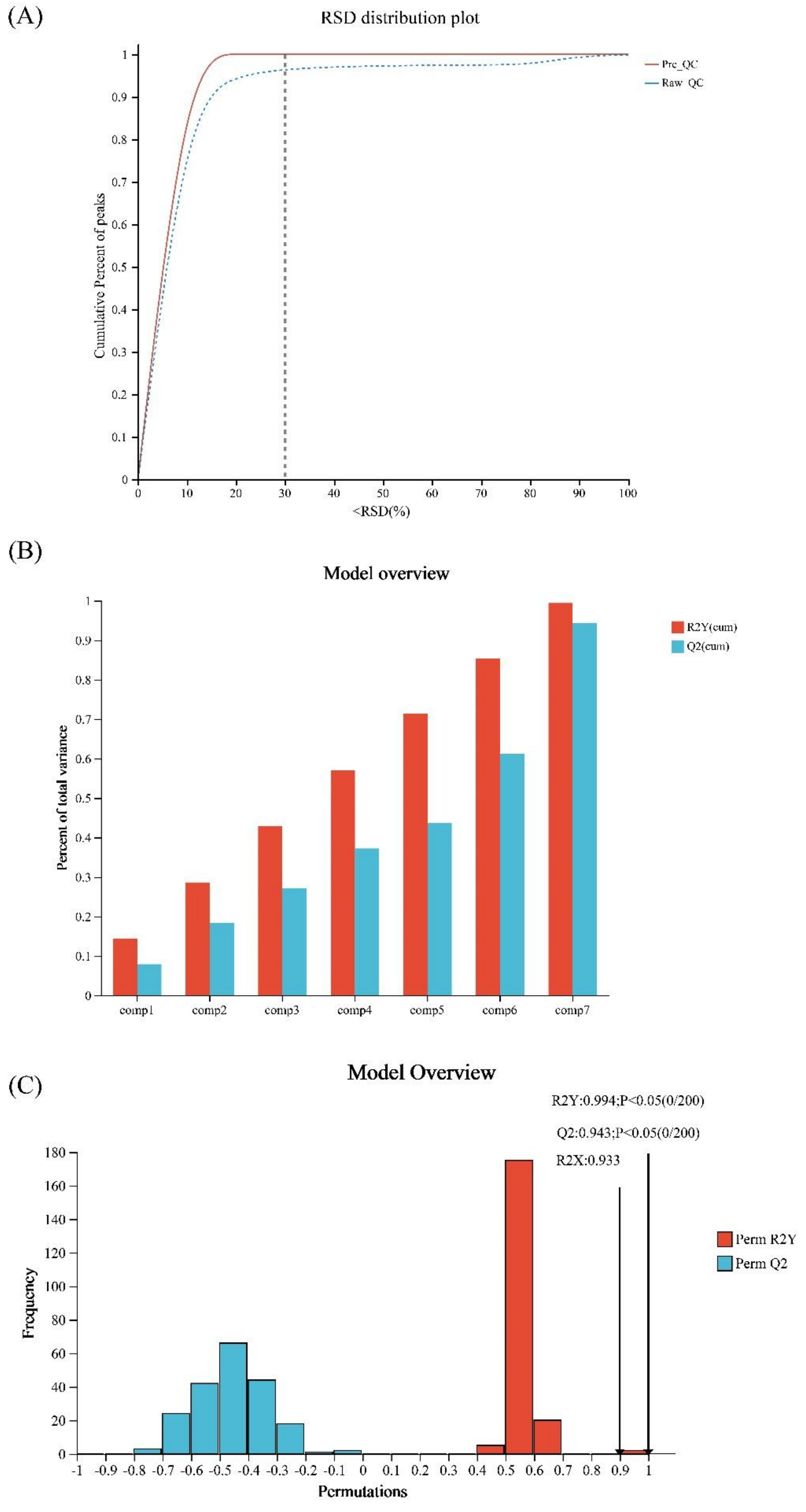

Figure S2. Metabolomics data quality and PLS-DA model validation. RSD distribution of QC samples (A). PLS-DA model cross-validation (B). Permutation test results of the PLS-DA model (C).

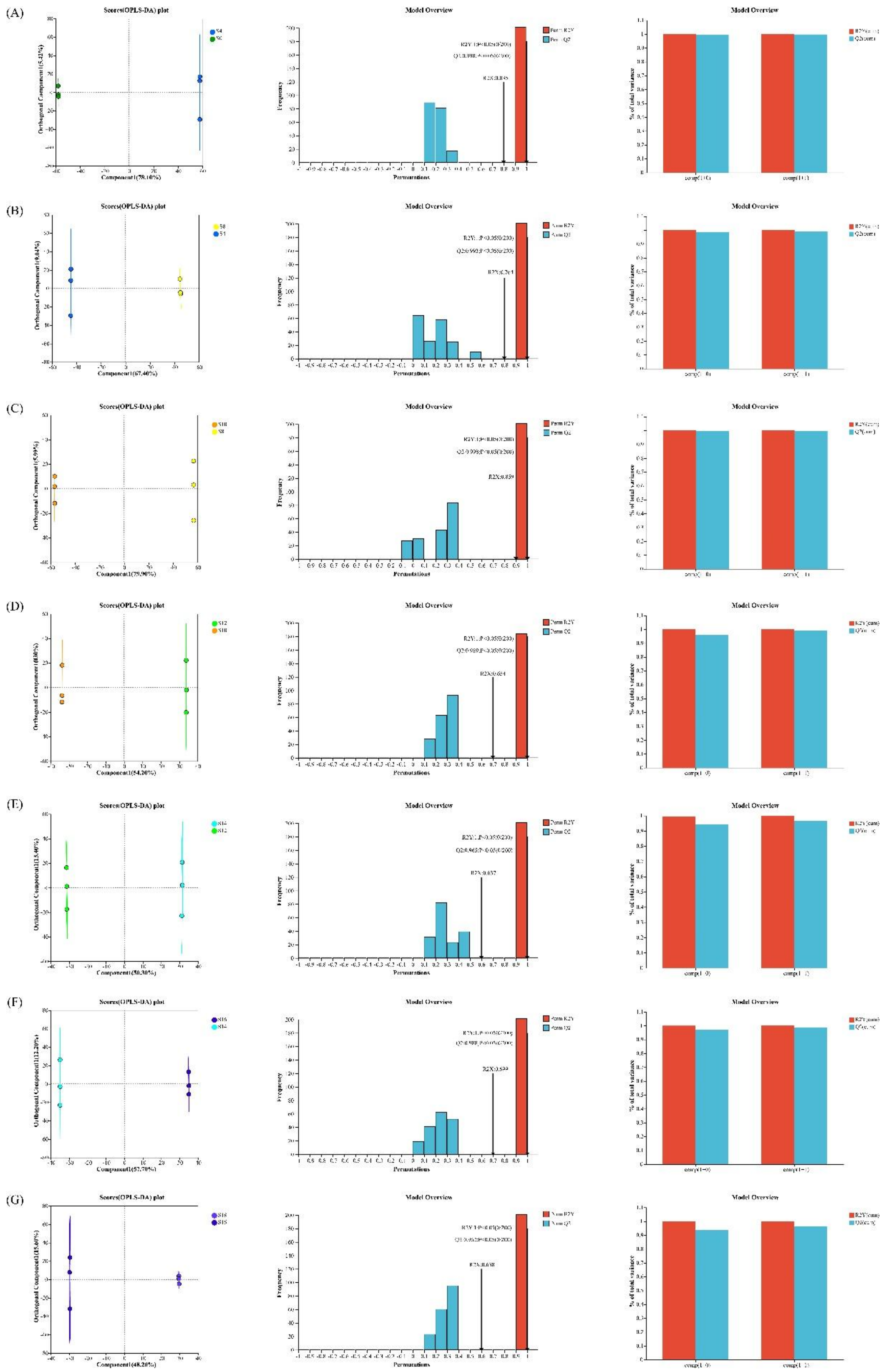

Figure S3. OPLS-DA models for pairwise comparisons during fermentation. Score plots and corresponding validation results for comparisons between adjacent sampling time points: S4 vs. S0 (A), S8 vs. S4 (B), S10 vs. S8 (C), S12 vs. S10 (D), S14 vs. S12 (E), S16 vs. S14 (F), S18 vs. S16

(G).
